# Adaptive stress response genes associated with breast cancer subtypes and survival outcomes reveal race-related differences

**DOI:** 10.1038/s41523-022-00431-z

**Published:** 2022-06-13

**Authors:** Muthana Al Abo, Larisa Gearhart-Serna, Steven Van Laere, Jennifer A. Freedman, Steven R. Patierno, Eun-Sil Shelley. Hwang, Savitri Krishnamurthy, Kevin P. Williams, Gayathri R. Devi

**Affiliations:** 1 Duke Cancer Institute, Duke University School of Medicine, Durham, NC 27710 USA; 2 Department of Surgery, Duke University School of Medicine, Durham, NC 27710 USA; 3 Center for Oncological Research (CORE), Faculty of Medicine and Health Sciences—University of Antwerp, Campus Drie Eiken‑Universiteitsplein 1, 2610 Wilrijk‑Antwerp, Belgium; 4 Department of Medicine, Division of Medical Oncology, Duke University School of Medicine, Durham, NC 27710 USA; 5 Department of Pathology, MD Anderson Cancer Center, Houston, TX 77030 USA; 6 Department of Pharmaceutical Sciences and BRITE, North Carolina Central University, Durham, NC 27707 USA

**Keywords:** Breast cancer, Population genetics, Prognostic markers, Breast cancer

## Abstract

Aggressive breast cancer variants, like triple negative and inflammatory breast cancer, contribute to disparities in survival and clinical outcomes among African American (AA) patients compared to White (W) patients. We previously identified the dominant role of anti-apoptotic protein XIAP in regulating tumor cell adaptive stress response (ASR) that promotes a hyperproliferative, drug resistant phenotype. Using The Cancer Genome Atlas (TCGA), we identified 46–88 ASR genes that are differentially expressed (2-fold-change and adjusted *p*-value < 0.05) depending on PAM50 breast cancer subtype. On average, 20% of all 226 ASR genes exhibited race-related differential expression. These genes were functionally relevant in cell cycle, DNA damage response, signal transduction, and regulation of cell death-related processes. Moreover, 23% of the differentially expressed ASR genes were associated with AA and/or W breast cancer patient survival. These identified genes represent potential therapeutic targets to improve breast cancer outcomes and mitigate associated health disparities.

## Introduction

According to the American Cancer Society (ACS)^1^, in recent years (2012–2016) there has been a continuous decline in the breast cancer death rate. Despite this, at the global level, notable differences in breast cancer mortality is observed among ethnic groups including younger age of onset^2,3^. These race-related disparities are likely driven by a complex interplay among sociocultural differences in societal-level (e.g., racism), neighborhood-level (e.g., pollution), and institutional-level (e.g., access to care) determinants of health^4^. Analysis of The Cancer Genome Atlas (TCGA) dataset adjusted for intrinsic subtype frequency differences has reported that patients estimated to have >50% African ancestry exhibit a worse breast cancer-free interval compared to patients with >50% European ancestry^5^. Underlying these ancestry-related disparities are likely individual-level differences in physiology, genetics and genomics arising from forced and voluntary migrations of human populations around the world. Further interactions among societal-level, neighborhood-level, institutional-level and individual-level determinants of health also likely contribute to cancer disparities by influencing allostatic load^6^.

Emerging evidence indicates that this race-related survival gap is largely due to higher incidence of aggressive subtypes of breast cancer, including basal-like, triple negative, human epidermal growth factor receptor 2 (ERBB2/HER2)-enriched subtypes, which are frequently associated with early metastasis in AA compared to W patients^7^. A highly representative example of an aggressive breast cancer designated as a cancer health disparity by NIH (NCATS/GARD)^8^ is inflammatory breast cancer (IBC). Of all clinically distinct breast cancer subtypes, IBC is the most lethal variant with high rate of metastasis, disproportionately higher incidence at younger ages in non-W patients, and disparity in relative 5-year survival rate ranging from 29.9 to 42.5% (higher survival outcomes in W patients)^9,10^.

AA patients with IBC present more frequently with higher stage and triple-negative (TN) [i.e., negative for estrogen (ER), progesterone receptor (PR), and human epidermal growth factor receptor 2 (ERBB2)] or basal subtype [similar to triple negative, but with epidermal growth factor receptor 1 (EGFR) activation]^11^, and exhibit shorter median survival (20 months) compared with W patients (32 months)^12–16^. Epidemiological studies suggest a distinct profile of risk factors such as high body mass index (BMI), early age at first pregnancy, multiparity, and lack of breastfeeding, factors that can lead to chronic pro-inflammatory cellular stress to be associated with poor therapeutic outcomes and survival in AA IBC patients compared with W patients^11,17^. Furthermore, comparative gene expression studies from preclinical models and pretreatment patient samples, collected as part of the International IBC Consortium’s effort to understand differences between IBC and non-IBC and to define IBC-specific molecular profiles, revealed highly activated mitogen activated protein kinase (MAPK) and nuclear factor kappa B (NFκB) transcriptional profiles associated with increased pro-inflammatory and proliferative signals in IBC compared with subtype and stage-matched locally advanced breast cancer^18–22^.

Preclinical studies using various breast cancer in vitro and in vivo models^23–28^ from our group identified a critical role for the most potent caspase inhibitor, X-linked inhibitor of apoptosis protein (XIAP), in linking EGFR-mediated MAPK activation and NFκB hyperactivity. In addition, higher XIAP staining was observed in invasive breast cancers compared to normal, benign ductal carcinoma in situ (DCIS), and higher XIAP also correlated with poor event free survival and increased lymph node involvement^29,30^. Importantly, XIAP has a unique element called an internal ribosomal entry sequence (IRES) in its 5’ untranslated region, which is critical for XIAP protein translation during response to cellular stress^31^. Using triple-negative cell lines, novel isotype-matched clonal isolates of tumor cells surviving exposure to acute/chronic stress stimuli, and genetically modified breast cancer cell variants with differential XIAP expression, we reported that XIAP upregulation allows tumor cells to survive in the presence of stressors like oxidative-^24,28^ and immune-mediated^27,32^ cell death stimuli, leading to clonal outgrowth of multi-drug resistant tumor cell populations^26,33^. Analysis of XIAP-overexpressing tumors exhibiting the characteristics of an adaptive stress response (ASR) revealed a dominance of proliferative, invasive, and immunosuppressive networks of NFκB target genes, which we term the “adaptive stress response (ASR) gene set”. This is highly relevant, as NFκB is recognized as a crucial mediator of inflammatory, immune, anti-apoptotic, and antioxidant signals as well as an important modulator of cancer stem cell biology, tumor surveillance, and tumor rejection^34^. The genes in the ASR gene set have also been reported to be part of an IBC-specific 79-gene signature enriched with activated gene networks in immune pathways and the TGFβ pathway that captured nearly 25% of IBC patient samples identified in the TCGA database as IBC-like^35,36^.

In the present study, we investigated the expression profile of the XIAP-driven ASR gene set in breast cancer subtypes, identified race-related differentially expressed genes within this gene set, and determined associations between ASR gene expression and poor survival outcomes.

## Results

### Proportion of subtypes, AA, and W patient samples in the TCGA breast cancer dataset

**Fig. 1:**
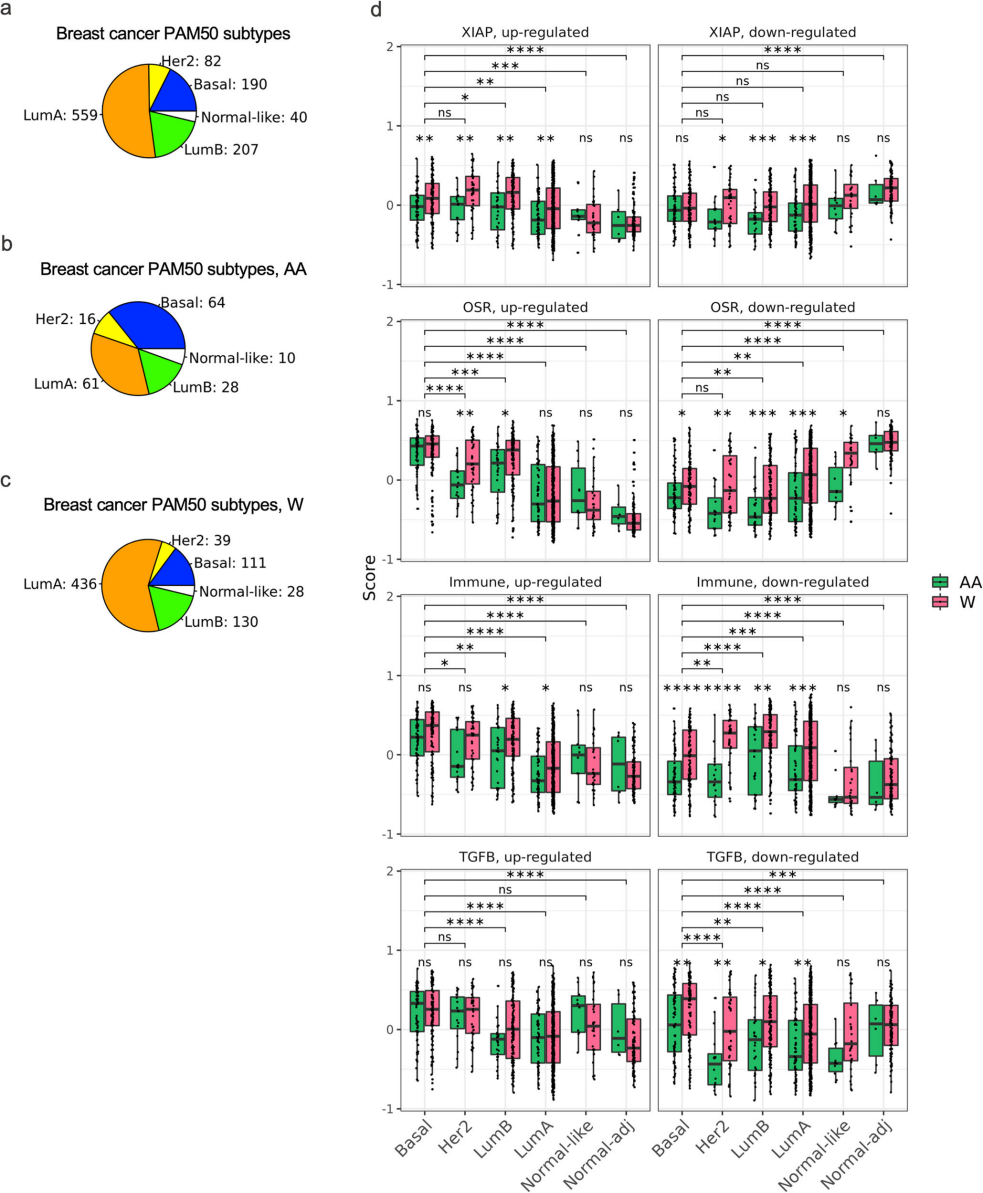
Number of breast cancer samples in TCGA by definition, race, and PAM50 subtypes. Pie charts depicting the number of breast cancer samples in TCGA PAM50 subtypes: Basal, Her2, LumB, LumA, Normal-like for all samples (**a**), for samples from AA patients (**b**), or for samples from W patients (**c**). **d** Scores of the expression level of XIAP, OSR-, Immune-, and TGFβ-related gene signatures in breast cancer subtypes. Box plots overlaid with scatter plots depicting the calculated score for XIAP-, OSR-, immune-, and TGFβ-related ASR gene signatures in the indicated breast cancer subtypes and stratified by patient race, AA or W. The Wilcoxon signed-rank test was used to examine significance (*adjusted *p*-value < 0.05; **adjusted *p*-value < 0.001; ***adjusted *p*-value < 0.0001; and ns, not significant). The center line represent the medians and the bounds of box represent the confidence intervals.

As of June 15, 2021, TCGA repository included 1222 breast cancer patient samples (1109 tumor and 113 matched normal). Furthermore, TCGA includes the Prosigna Prognostic Gene Signature Assay (formerly called the PAM50 test) to categorize breast tumors into five subtypes [luminal A (LumA), luminal B (LumB), Her2, Basal, and Normal-like] based on the expression level of genes, which have been found to be associated with breast cancer prognosis. In the present study, we focused on primary tumor samples [1090 Primary Solid Tumor] and normal tissue samples adjacent to the tumors [113 Solid Tissue Normal] (referred to in the present study as Normal-adjacent). Within these, our sample set included 559 lumA, 207 lumB, 82 Her2, 190 Basal, and 40 Normal-like (Fig. 1a; Supplementary Table 1). Metastatic samples and those lacking PAM50 subtype information were excluded. When more than one sample belonged to the same patient, we selected the one with highest RNA sequencing depth.

Racial designations in TCGA are based on patient self-identification. In the present study, race-related analysis focused on AA and W breast cancer patient datasets from TCGA (179 AA and 744 W). It is important to note that among the 113 Normal-adjacent samples, there are 105 W and only 6 AA samples. PAM50 subtyping within the AA and W patient tumor datasets are shown in Fig. 1b, c; Supplementary Table 1.

### ASR genes associated with oxidative stress and immune response pathways score highly in basal breast cancer subtype

To build and expand on our previous analysis of IBC tumor cells surviving under chronic stress stimuli, which identified genes in the anti-apoptotic signaling, oxidative stress response, immune, and TGFβ-related genes, we calculated the gene signature scores for the above pathways in the TCGA breast cancer subtypes and by patient race, AA and W (Fig. 1d). The gene sets are listed in Table 1 and are part of the overarching ASR gene set. We sorted the genes in each signature into up- or downregulated genes according to the expression levels reported in our previous preclinical studies^23,30,35,37^. The results from this analysis demonstrated that the score of ASR gene signatures differed among breast cancer subtypes and by patient race. We found that the XIAP downregulated gene set exhibits differential score between AA and W patients in LumA, LumB, and Her2 subtypes, and the XIAP upregulated gene set exhibits differential score between AA and W patients in Basal, Her2, LumB, and LumA subtypes. The differential score of OSR signature is highest in Basal, followed by Her2, and LumB, and lowest in Normal-like subtype, compared with Normal-adjacent. Comparing the OSR signature scores by patient race indicated that scores differed in the breast cancer subtypes between samples from AA and W patients, especially for the OSR downregulated genes. The score analysis also showed that immune-related upregulated genes generally exhibited higher scores in tumors compared to normal. However, the immune-related downregulated genes did not exhibit a distinct pattern in different tumor subtypes. Importantly, immune-related downregulated gene expression in Her2 and Basal subtypes showed significant differences (adjusted *p*-value < 0.0001) between AA and W patients. We also observed that the score of TGFβ-related downregulated genes in Her2 subtype exhibited a marked difference between AA and W patients.

**Table 1:** The list of ASR genes and their functional pathways.

Pathway	ASR genes
XIAP	*XIAP, SPANXA1, SPANXB1, SPANXC, SULT1E1, SLPI, AGFG2, POPDC3, ZIC1, CDC45, TNFSF9, SLC26A6, KYNU, H2BC11, MCM5, GINS2, CDKN2D, OBSL1*, ***SNAI1****, TP53I3, AIM2,* ***LIG1****, PIMREG, ALDOC*, ***MCM10****,****ASF1B****, ZNF165, CDKN1C, H2AC8, TMEM40, DBP, SLCO1B3,* ***E2F8****, LRP4, KLRC1, RALGDS*, ***BLM****,* ***CCNE2****,* ***POLE2****, ZNF26, PITPNC1, NEU1, VASH2, PLAU, GLYR1, H2BC21, NRN1, TBC1D3, COPZ2, H2BC8, KLRC2, RPL5*, ***SEPTIN6****, CRYBG3, PRR16, WDR37, TBC1D3C, FOSB, ASTE1, CLCN6, PDE4DIP, N4BP2L1, LEF1, HSPG2, POLA1, WBP1L, ZMYM5, B3GNT2*, ***DHFR****, ZNF331, ACSM3, MANSC1, ARAP2, TMEM100, SLC1A4, GPR137B, LIPG, LMAN1, LPAR6, SNX19, CYP4B1, GEM, ARHGAP29, ZFPM2, SLC14A1, PCDH7, HSD11B1, CAVIN2, ABAT, STC1, NRIP3, NAT1, PCDH9, LMAN2L, REXO5, DLEU2L, DLEU2, SYBU*, ***MRM2****, LINC-PINT, LINC00115*
OSR	*HSPA1A, METTL7A*, ***BLM****, TYMS, KRT6B*, ***LIG1****, SCD5,* ***POLE2****, CDT1,* ***CCNE2****, SKP2*, ***E2F8****, ORC1, MCM4, CCN3, IRF4*, ***MCM10****, NAP1L3, MCM3*, ***ASF1B****, FGF2, TREM1, ABCA6, CTH, ECM2, OASL, CEBPD, TP63, RAB5A, SLC4A7, MBNL2, RBMS3, KLHL24, TXNIP, FCAR, AREG, ANXA3, PTGS2, NRG1, INHBE*
NFkB	*NFKB1, RELA*, ***IL6****, CXCL8, IRF2, FAS*, ***IL1B****, BCL2, BIRC5, SOD1, MYC*
MNK	MKNK1, MKNK2, EIF4G1, EIF4E, SPRY2, HNRNPA1, HNRNPA2B1, NONO, KAT5, RPS6
Immune	*CTSA, PNP, MYCBP2, TMC6, IFI44L, NUDT1, MELK, STAT4, INHBC, ARPC2, EHD1, SART3, MBD4, ACOX1, PRKCB, NFATC3, FOLR1, MAK16, WNK1, JMJD6, IQGAP1, ABCC10, SRSF7, IGLV1-44, NUP85*, ***SEPTIN6****, ESF1, NAA15, PGS1, ANKRD11, TNPO1, PAX5*, ***DHFR****, CTBP2, BCKDK, PEX11B, CUL2, GABPB1, ATP7A, ATF2, DNAJB6, CANX, PAK2, ANXA7, TMCO1, PBXIP1, SP3*, ***TGIF2****, TOMM22, INTS12, SLC30A5, TMEM50B, TSPAN14, TBL1XR1*, ***MRM2***
TGFβ	*TGFB1, NDUFAF3, DAB2, RPL27A, MARCKS, CD72, HSP90B1, PPARD, ACTG1, CRK, TGFB2, AUTS2, RYK*, ***TGIF2***
JAG1-Notch	*JAG1, NOTCH1, DLL4, BRD4*, ***IL6***, ***IL1B****, TNF*, ***SNAI1****, ZEB1*

The ASR genes were grouped according to their function in the listed pathways. The ASR genes that are bolded represent the genes belonging to more than one of the indicated pathways.

### Identification of differential ASR gene sets in PAM50 subtypes

**Fig. 2:**
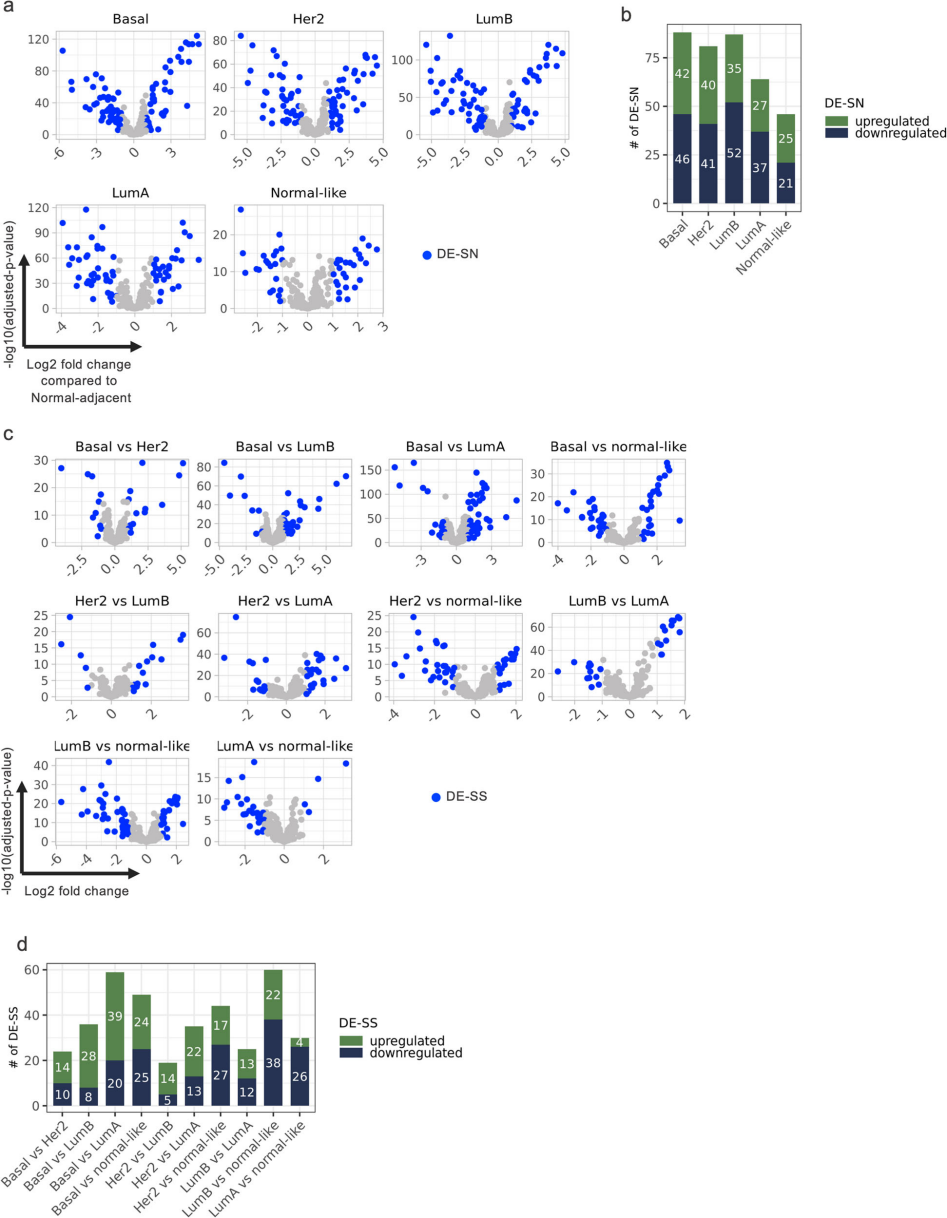
Identification of DE-SN and DE-SS in PAM50 subtypes. Volcano plots depicting the level of ASR genes in the indicated PAM50 subtypes compared to Normal-adjacent (**a**) or to each other (**b**). The log2 fold-change differential expression and the –log10 (adjusted *p*-value) are shown on the *x*-axis and *y*-axis, respectively. The points correspond to all ASR genes and the blue highlighted points represent the DE-SN (**a**) and DE-SS (**c**), which exhibit fold-change greater than 2 and adjusted *p*-value less than 0.05. **b** and **d** Bar plots depicting the number of DE-SN and DE-SS in **a** and **c**, respectively. The colors of the bars indicate whether the DE-SN (**b**) or DE-SS (**d**) are upregulated (green) or downregulated (blue). The numbers inside the bar correspond to the number of upregulated or downregulated DE-SN (**b**) or DE-SS (**d**). Comparisons are shown under each bar and the number of DE-SN (# of DE-SN) or DE-SS (# of DE-SS) are shown on the *y*-axis.

To identify the expression levels (combined level of all transcript variants of any given gene) of the adaptive stress response (ASR) gene sets (Table 1) in relation to PAM50 subtypes, we performed analyses comparing gene expression in samples of each PAM50 subtype with Normal-adjacent samples (Fig. 2a). We determined differentially expressed genes between PAM50 subtypes compared with Normal-adjacent (designated DE-SN) with > 2-fold-change in mean expression and adjusted *p*-value < 0.05. In total, we identified 88 DE-SN in Basal, 81 in Her2, 87 in luminal B, 64 in luminal A and 46 in Normal-like samples (Fig. 2b and Supplementary Table 2). We also determined the number of upregulated and downregulated DE-SN in each subtype.

Similarly, to identify differentially expressed genes within ASR gene sets between subtypes, we compared expression levels of the 226 genes in each PAM50-subtype to every other subtype (designated DE-SS). This analysis revealed 24 DE-SS in Basal vs. Her2, 36 in Basal vs. LumB, 59 Basal vs. LumA, 49 Basal vs. Normal-like, 19 Her2 vs. LumB, 35 Her2 vs. LumA, 44 Her2 vs. Normal-like, 25 LumB vs. LumA, 60 LumB vs. Normal-like, and 30 LumA vs. Normal-like samples (Fig. 2c, d and Supplementary Table 3). Collectively, this analysis identified upregulated or downregulated DE-SS in a given subtype compared to the other subtype in each comparison.

We noted differences between DE-SN and DE-SS gene sets. In the case of DE-SN, an equal number of genes (about half) were either upregulated or downregulated compared with Normal-adjacent, regardless of the tumor subtypes (Fig. 2b). In contrast, the number of upregulated or downregulated DE-SS varied depending on the comparison between subtypes (Fig. 2d). For example, DE-SS in LumA vs. Normal-like were more frequently downregulated (26 downregulated and 4 upregulated). In contrast, DE-SS in Basal vs. LumA tended to be upregulated (39 upregulated and 20 downregulated).

The proportion of DE-SN among the 226 genes was 69.4% when comparing subtypes to Normal-adjacent samples and 34.8% when comparing subtypes to each other. As a control, we identified DE-SN and DE-SS among 226 randomly selected genes. Unlike the ASR gene set, the proportion of DE-SN was less than 3% when comparing subtypes to Normal-adjacent samples and the proportion of DE-SS was less than 3% when comparing subtypes to each other.

### DE-SN and DE-SS exhibit distinct expression changes in breast cancer molecular subtypes

**Fig. 3:**
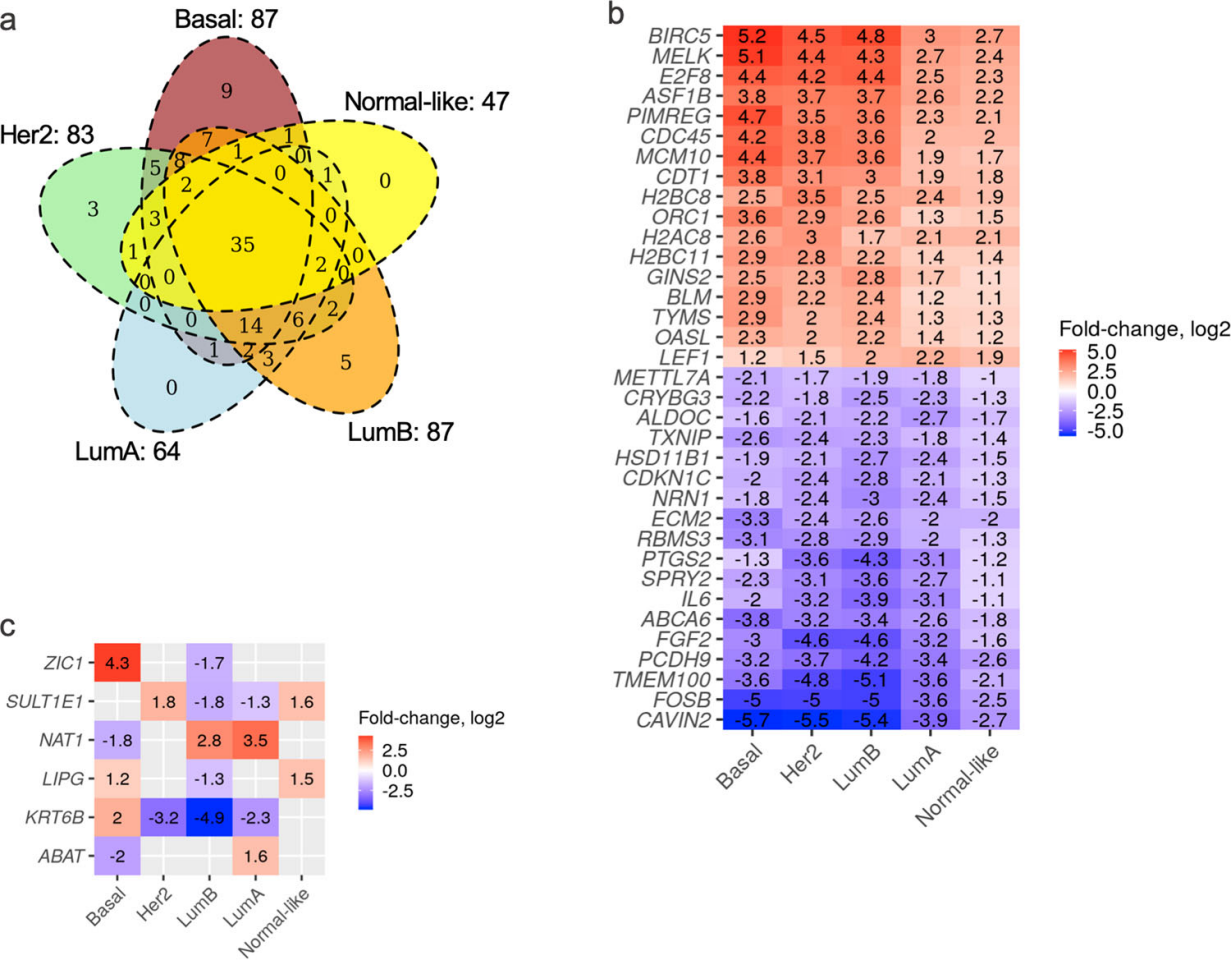
Intersection among DE-SN. **a** Venn diagrams depicting the common DE-SN after comparing PAM50 subtypes to Normal-adjacent samples. **b** and **c** Heatmaps depicting the expression fold-change for DE-SN that are significantly changed in all PAM50 subtypes (**b**) or for DE-SN that are dynamically differentially expressed across PAM50 subtypes (**c**).

Next, we investigated whether DE-SN are shared among subtypes. The vast majority of DE-SN are differentially expressed in more than one subtype, with only a few of the DE-SN being unique to a particular subtype (Fig. 3a). Thirty-five DE-SN were identified as being commonly differentially expressed across all subtypes compared with Normal-adjacent samples (Fig. 3b and Supplementary Table 2). We did not find any tendency toward enrichment in upregulated or downregulated commonly differentially expressed genes across all subtypes, as 17 DE-SN were upregulated and 18 were downregulated among all subtypes compared with Normal-adjacent (Supplementary Fig. 1a, b).

Expression levels of a number of DE-SN fluctuate dynamically among different subtypes (Fig. 3c). For example, *4-Aminobutyrate aminotransferase* (*ABAT*) (member of XIAP pathway/Table 1) expression level was decreased 4-fold in Basal subtype compared with Normal-adjacent but increased 3-fold in LumA compared with Normal-adjacent. We also found other XIAP pathway genes, *Arylamine N-acetyltransferase* (*NAT1*), *Lipase G* (*LIPG*), *Sulfotransferase Family 1E Member 1* (*SULT1E1*), and *Zic Family Member 1* (*ZIC1*) genes and *Keratin 6B* (*KRT6B*) (member of the OSR pathway/Table 1) that were differentially expressed with varying magnitudes depending on the subtype (Fig. 3c).

### Identification of distinct DE-SN and DE-SS in AA and W breast cancer patients

**Fig. 4:**
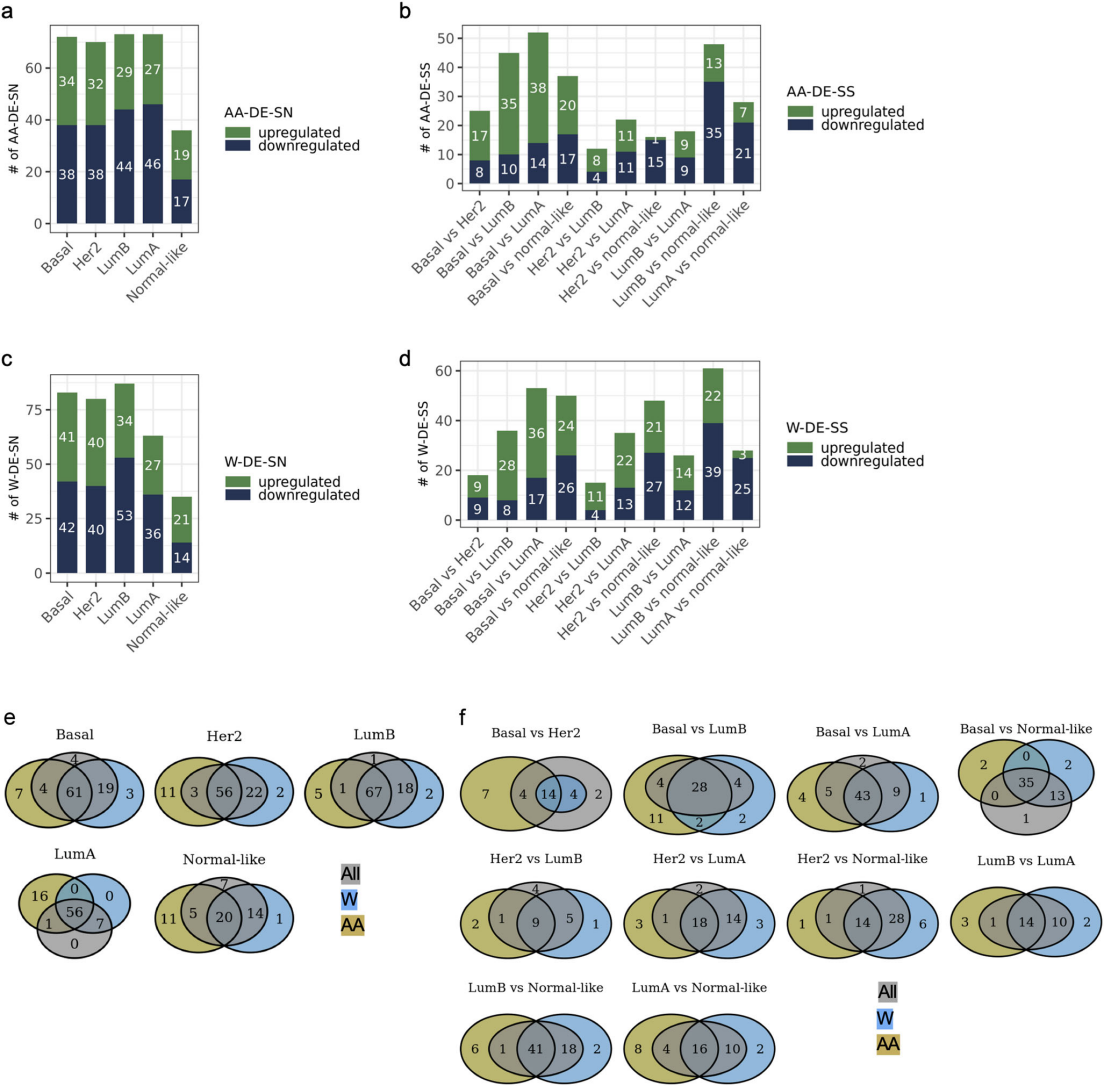
Identification of DE-SN or DE-SS in PAM50 subtypes from either AA or W patients. Bar plots depicting the number of DE-SN (**a**) or DE-SS (**b**) in breast cancer samples from AA patients. Similar to **a** and **b**, **c** and **d** depict the number of DE-SN or DE-SS in breast cancer samples from W patients, respectively. The colors of the bars, the numbers inside the bars, and the axes are as described in Fig. 2. **b** As in **a**, but the DE-SS are identified after comparison of PAM50 subtypes to each other. Intersection among DE-SN (**e**) or DE-SS (**f**) with or without stratification of samples by patient race. **e** and **f** Venn diagrams depicting the common DE-SN identified after comparison between samples of the indicated PAM50 subtype with Normal-adjacent samples or after comparison between samples of the indicated PAM50 subtypes, respectively. Light blue circles represent the DE-SN identified without stratification by patient race, the light purple circles represent the DE-SN identified among breast cancer samples from W patients only and the yellow circles represent the DE-SN identified among breast cancer samples from AA patients only.

Interrogating the differential expression of the ASR gene sets in breast cancer TCGA samples from AA or W patients has the potential to identify key biological factors that contribute to breast cancer disparities. Therefore, we compared the expression of the 226 genes in PAM50 subtypes to Normal-adjacent or to other subtypes in either samples from AA patients only or from W patients only (AA-DE-SN, AA-DE-SS, W-DE-SN, W-DE-SS, respectively) (Fig. 4 and Supplementary Tables 4 and 5). The number of AA-DE-SN ranged from 36 to 71 and the number of AA-DE-SS ranged from 12 to 52 (Fig. 4a, b). The number of W-DE-SN ranged from 35 to 87 and the number of W-DE-SS ranged from 15 to 61 (Fig. 4c, d). Although the majority of the DE-SN or DE-SS identified among all samples, samples from AA patients only, or samples from W patients only overlapped, a number of AA- or W-DE-SN and AA- or W-DE-SS were only identified after race stratification (Fig. 4e, f). Therefore, importantly, identification of a number of DE-SN and DE-SS are race-related. For example, in Basal subtype, we identified 88 DE-SN, 72 AA-DE-SN and 81 W-DE-SN. There were 61 overlapping genes among DE-SN, AA-DE-SN, and W-DE-SN, 7 genes specific to samples from AA patients only, and 3 genes specific to samples from W patients only. There were only 4 shared genes between DE-SN and AA-DE-SN compared to 19 shared genes between DE-SN and W-DE-SN in Basal subtype (Fig. 4e). The genes that are specifically differentially expressed as part of ASR pathways studied (Table 1) in Basal tumors of AA patients are *Nucleoporin 85* (*NUP85*) (member of the immune pathway), *Solute Carrier Family 26 Member 6* (*SLC26A6*) (member of the XIAP pathway), *Cathepsin A* (*CTSA*) (member of the immune pathway), *ATPase Copper Transporting Alpha* (*ATP7A*) (member of the immune pathway), *TNF Superfamily Member 9* (*TNFSF9*) (member of the XIAP pathway), *Snail Family Transcriptional Repressor 1* (*SNAI1*) (member of the XIAP and JAG1-Notch pathways), *and Nuclear Receptor Interacting Protein 3* (*NRIP3*) (member of the XIAP pathway). The genes that are specifically differentially expressed in Basal tumors of W patients are *TGFB Induced Factor Homeobox 2* (*TGIF2*) (member of the immune and TGFβ pathways), *SPANX Family Member B1* (*SPANXB1*) (member of the XIAP pathway), and *Transmembrane Protein 40* (*TMEM40*) (member of the XIAP pathway).

### Identification of race-related differentially expressed ASR genes within PAM50 subtypes

Next, we investigated if any of the ASR genes exhibited race-related differential expression by comparing TCGA breast cancer samples of a given PAM50 subtype between AA and W patients. This analysis identified a number of race-related differentially expressed ASR genes, which included 1 in Basal, 7 in Her2, 4 in LumB, 3 in LumA, and 14 in Normal-like samples (Table 2). Among the identified race-related differentially expressed ASR genes, we found that *Crystallin Beta-Gamma Domain Containing 3* (*CRYBG3*) (member of the XIAP pathway/Table 1) is a race-related differentially expressed ASR gene in Her2 and LumA subtypes, *CXCL8* (member of the NFkB pathway/Table 1) and *Stanniocalcin 1* (*STC1*) (member of the XIAP pathway/Table 1) are race-related differentially expressed ASR genes in Her2 and normal-like subtypes, and *Transmembrane Protein 100* (*TMEM100*) (member of XIAP pathway/Table 1) is a race-related differentially expressed ASR gene in LumA and normal-like subtypes.

**Table 2:** Differentially expressed ASR genes within a given PAM50 subtype between AA or W.

	Symbol	Log2 FC AA - W	AverExp (log2)	*p*-value	Adjusted *p*-value	Function in cancer
Basal	*CEBPD*	1.06	4.97	1.4E^−10^	3.9E^−09^	ccaat/enhancer-binding protein δ, associates with good prognosis in breast cancer ^TR1^.
Her2	*CRYBG3*	−1.75	3.4	1.6E^−18^	3.0E^−16^	Long non-coding RNA, its depletion in tumor cells enhanced their malignant phenotypes ^TR2^.
	*TBC1D3*	1.17	−6.36	1.6E^−09^	3.5E^−08^	TBC1D3 oncogene promotes the migration of breast cancer cells ^TR3^.
	*CXCL8*	−1.56	1.07	1.8E^−02^	4.6E^−02^	Interleukin-8, promote breast cancer progression by increasing cell invasion, angiogenesis, and metastases and is upregulated in HER2-positive cancers ^TR4^.
	*STC1*	−1.36	5.34	2.0E^−06^	2.2E^−05^	Stanniocalcin-1, the role of STC1 in breast cancer is complex, considering that some studies have shown that it exerts an oncogenic role, whereas other studies have demonstrated the opposite ^TR5^.
	*CCNE2*	−1.03	2.63	2.0E^−03^	8.2E^−03^	Cyclin E2, associates with poorer prognosis in breast cancer ^TR6^.
	*TP63*	−1.7	2.66	1.5E^−04^	1.0E^−03^	p53-related protein p63. ΔNp63 isoform supports a more mesenchymal phenotype associated with a higher tumorigenic and metastatic potential ^TR7^.
	*H2AC8*	1.12	1.23	2.2E^−03^	8.9E^−03^	Expression of HIST1HSAE associated with disease free survival in colorectal cancer ^TR8^.
LumB	*SPANXA1*	1.02	−6.71	1.2E^−08^	2.3E^−07^	Sperm Protein Associated With The Nucleus, X-Linked, Family Member A1, promote breast cancer invasion^TR9^.
	*SPANXC*	1.41	−6.26	6.7E^−06^	6.8E^−05^	Same as *SPANXA1*.
	*NAT1*	−1.29	5.35	3.1E^−03^	1.2E^−02^	Arylamine N-acetyltransferase 1, a potential marker in estrogen receptor-positive tumors^TR10^.
	*SPANXB1*	1.37	−5.18	1.3E^−05^	1.2E^−04^	Sperm Protein Associated With The Nucleus, X-Linked, Family Member A1, promote breast cancer invasion^TR11^.
LumA	*CRYBG3*	−1.49	3.4	1.6E^−18^	3.0E^−16^	See above.
	*SPANXB1*	1.5	−5.18	1.3E^−05^	1.2E^−04^	Same as *SPANXA1*.
	*TMEM100*	−1.04	0.06	3.6E^−04^	2.2E^−03^	Transmembrane protein 100, Low-expression associates with poor prognosis in non-small-cell lung cancer^TR12^.
Normal-like	*FGF2*	−1.34	2.45	7.2E^−07^	9.0E^−06^	Fibroblast growth factor 2, induces breast cancer growth^TR13^.
	*CDT1*	1.34	3.71	1.8E^−12^	7.6E^−11^	Chromatin licensing and DNA replication factor 1, significantly higher in ER-negative breast cancer^TR14^.
	*NRG1*	−1.61	1.28	5.8E^−04^	3.4E^−03^	Neuregulin 1, frequently silenced by methylation in breast cancers^TR15^.
	*PTGS2*	−1.57	0.98	1.3E^−04^	9.7E^−04^	Prostaglandin-endoperoxide synthase 2, genetic variation in this gene is associated with breast cancer risk ^TR16^.
	*CRYBG3*	−1.13	3.4	1.6E^−18^	3.0E^−16^	See above.
	*BIRC5*	1.11	5.03	9.5E^−06^	9.2E^−05^	Baculoviral inhibitor of apoptosis repeat containing 5, high expression associates with poor survival ^TR17^.
	*CDC45*	1.41	3.01	3.0E^−07^	4.0E^−06^	Cell division cycle 45, higher expression in cancer cells and might associate with metastasis ^TR18^.
	*TNFSF9*	1.04	−0.25	3.9E^−03^	1.4E^−02^	Tumor necrosis factor superfamily member 9, an immune modulating T-cell co-stimulator with anti-tumor role ^TR20^.
	*PIMREG*	1.13	2.43	5.2E^−04^	3.1E^−03^	PICALM interacting mitotic regulator, overexpression promotes breast cancer aggressiveness ^TR21^.
	*CD72*	1.18	1.9	5.9E^−05^	4.7E^−04^	Potential role in anti-tumor immune response ^TR22^.
	*STC1*	−1.39	5.34	2.0E^−06^	2.2E^−05^	See above.
	*TMEM100*	−1.2	0.06	3.6E^−04^	2.2E^−03^	Transmembrane protein 100, inhibits the growth and metastasis of non-small-cell lung cancer ^TR23^.
	*CXCL8*	−1.39	1.07	1.8E^−02^	4.6E^−02^	See above.
	*ZFPM2*	−1.11	2.37	2.6E^−08^	4.5E^−07^	Zinc finger protein, FOG family member 2, linked to tumor stage, metastasis, and prognosis in breast patients ^TR24^.

Race-related differentially expressed ASR genes identified after comparing breast cancer samples of a given PAM50 subtype between AA and W patients. The fold-change (FC) and the average expression (AverExp) are shown.

### Gene ontology analysis of AA- and W-DE-SN reveals differential ontology enrichment

**Fig. 5:**
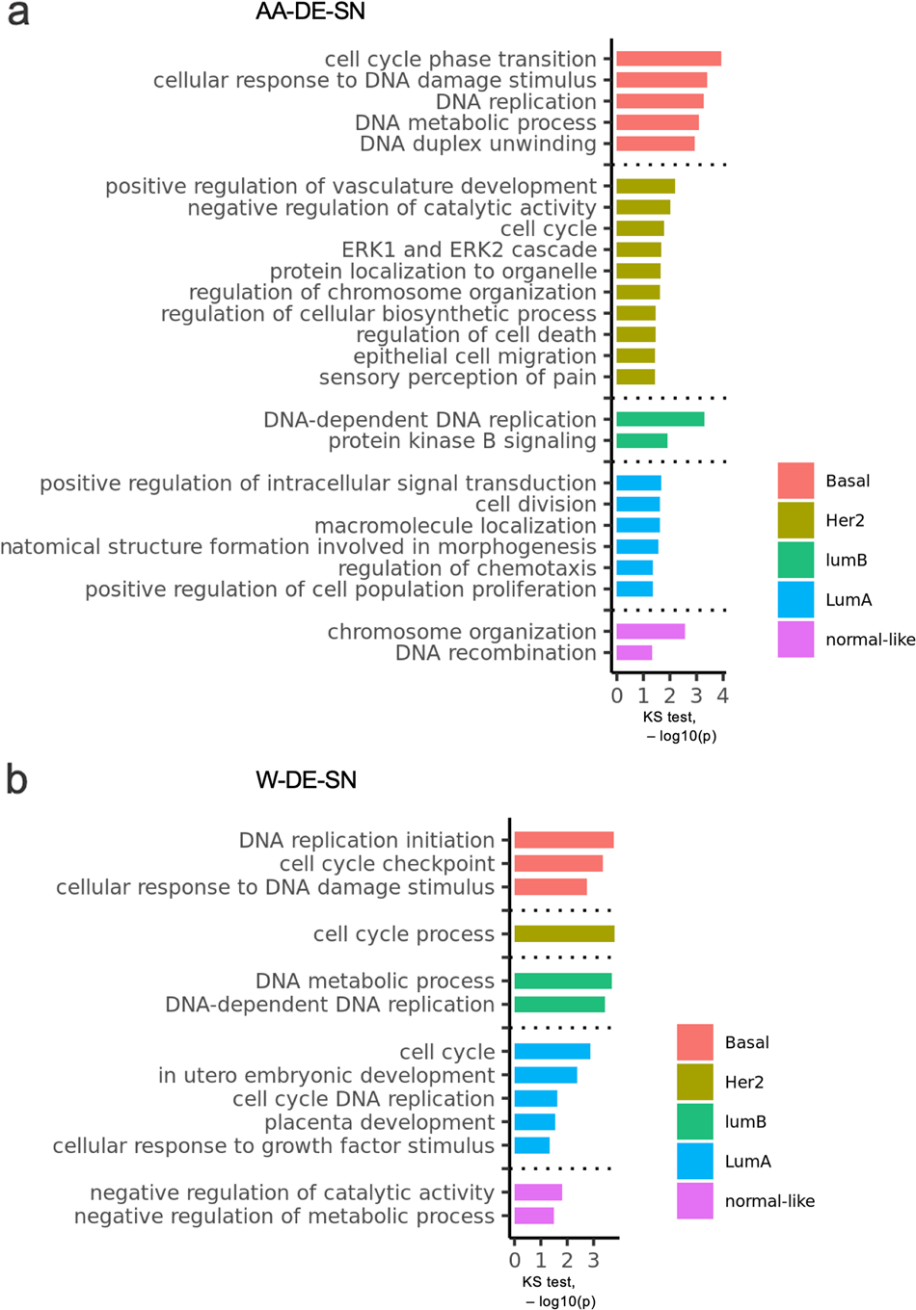
GO enrichment analysis of DE-SN. Bar plots depicting the significantly, *p*-value < 0.05, enriched GO terms of the DE-SN in PAM50 subtypes from either AA patients only (**a**) or from W patients only (**b**). The *x*-axis in **a** and **b** depicts the –log10 *p*-value yielded from the Kolmogorov–Smirnov test. The enriched GO terms are included next to the bars.

To understand the functions of the identified DE-SN in breast cancer samples from AA or W patients, we first queried for associated Gene Ontology (GO) categories and then submitted for GO enrichment analysis. This GO enrichment analysis of AA- or W-DE-SN in a given PAM50 subtype revealed GO enrichment in cell cycle, DNA damage response, signal transduction, and regulation of cell death processes (KS < 0.05) (Fig. 5a, b).

Notably, DNA replication, metabolism, and damage response processes were enriched in Basal and Her2 subtypes. We compared the enriched GOs for AA-DE-SN and W-DE-SN within each subtype and found differential GO enrichment, especially in Her2 and Normal-like subtypes. For example, the regulation of cell death, ERK1 ERK2 cascade, and epithelial cell migration processes were enriched among AA-DE-SN in the Her2 subtype, but not among W-DE-SN in the Her2 subtype, and the DNA recombination and chromosomal organization processes were enriched among AA-DE-SN in the Normal-like subtype, but not among W-DE-SN in the Normal-like subtype. The functional annotation of race-related DE highlights their potential function in oncogenesis and are included in Table 2.

### Association of DE-SN with breast cancer patient survival

**Fig. 6:**
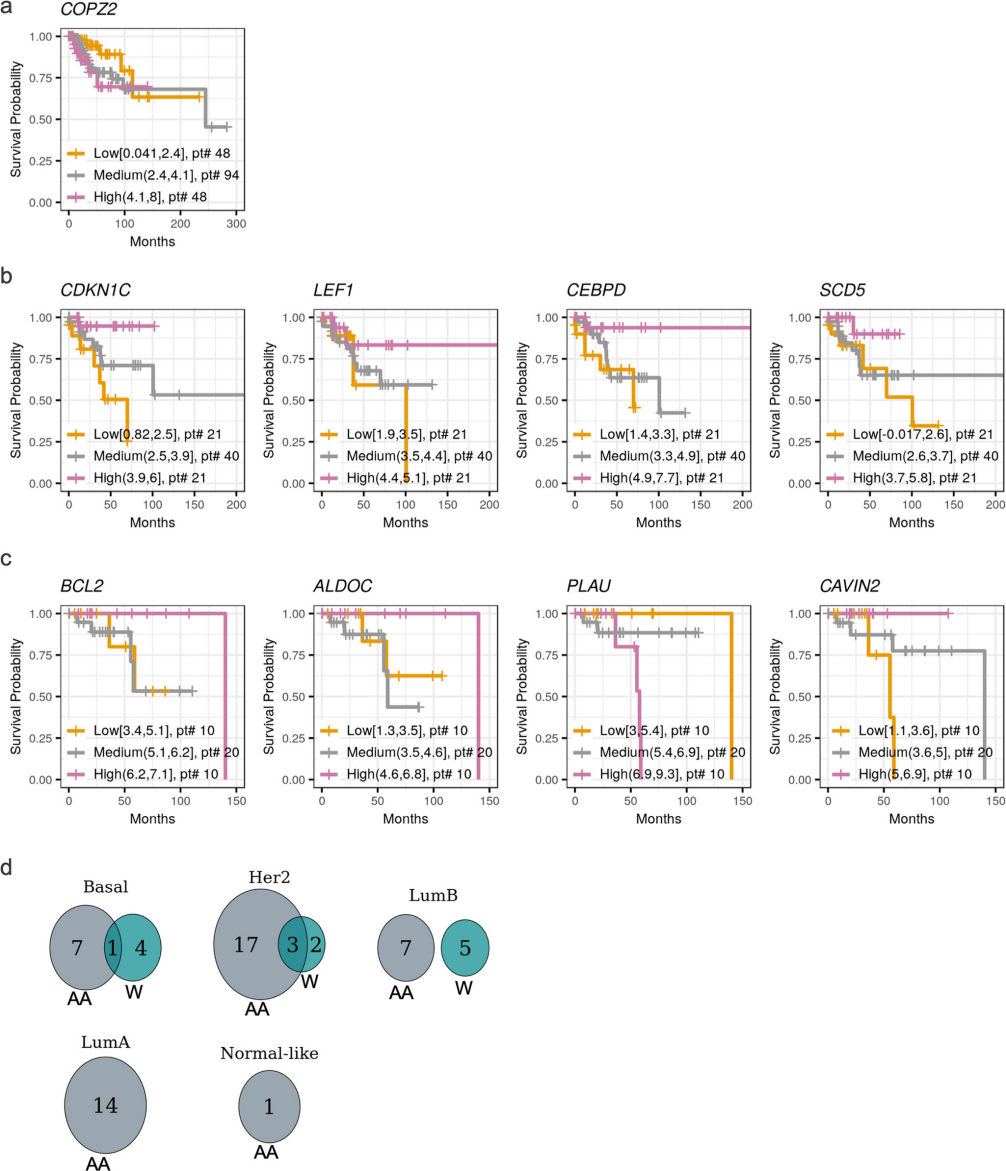
Survival analysis of the DE-SN. Kaplan–Meier plots for the DE-SN depicting the association of DE-SN level in Basal (**a**), Her2 (**b**), and Normal-like (**c**) with breast cancer patient overall survival probability. The DE-SN exhibit HR > 1.5 or < 0.58 and *p*-value < 0.05. The survival probabilities were compared in breast cancer patients of indicated PAM50 subtypes expressing high, low, or intermediate (75th, 25th, or 25th–75th percentiles) levels of the indicated DE-SN The number of patients (pt#) of high, low, or intermediate groups are indicated. **d** Venn diagrams showing the number of DE-SN associated with AA or W breast cancer patient overall survival probability in the indicated PAM50 subtypes.

To gain insight into the potential clinical relevance of race-related DE-SN identified, we performed overall survival analysis using TCGA clinical data. The expression levels of DE-SN and patient survival data were fitted into Cox regression models to compute the hazard ratio (HR) for each DE-SN. Survival data of all patients of each PAM50 subtype (excluding ones with metastases), were included in the survival analysis. In all our analyses, a given DE-SN was significantly associated with patient overall survival if HR > 1.5 or HR < 0.58 and *p*-value < 0.05. As shown in Fig. 6a–c, a number of DE-SN were associated with the survival of all patients (regardless of patient race), as follows: *COPI Coat Complex Subunit Zeta 2* (*COPZ2*) in Basal; *Cyclin Dependent Kinase Inhibitor 1C* (*CDKN1C*); *LEF1*, *CCAAT/enhancer-binding protein delta* (*CEBPD*), *Stearoyl-CoA desaturase* (*SCD5*) in Her2; and *BCL2 Apoptosis Regulator* (*BCL2*), *Aldolase, Fructose-Bisphosphate C* (*ALDOC*), *Plasminogen Activator, Urokinase* (*PLAU*) and Caveolae Associated Protein 2 (*CAVIN2*) in Normal-like samples. The above genes listed are all members in the XIAP pathway except *BCL2* (member of NFkB pathway), *CEBPD* and *SCD5* (both members of the OSR pathway).

Next, we investigated the association between DE-SN and overall survival within each breast cancer subtype stratified by race (HR > 1.5 or HR < 0.58 and *p*-value < 0.05). From this analysis, we identified the following race-related survival associations: among the 88 DE-SN in Basal subtype, 7 DE-SN associated with survival among AA patients, 4 among W patients, and 1 among both AA and W patients; among the 81 DE-SN in Her2 subtype, 17 DE-SN associated with survival among AA patients, 2 among W patients, and 3 among both AA and W patients; among the 87 DE-SN in LumB subtype, 7 DE-SN associated with survival among AA patients and 5 among W patients; among the 64 DE-SN in LumA subtype, 14 DE-SN associated with survival among AA patients; and among the 46 DE-SN in Normal-like subtype, 1 associated with survival among AA patients (Fig. 6d and Table 3). Of particular interest in the context of breast cancer disparities are the DE-SN whose levels were specifically associated with AA breast cancer patient overall survival, which included members of XIAP and OSR pathway [*BLM RecQ Like Helicase* (*BLM*) and *E2F Transcription Factor 8* (*E2F8*)] in Basal; the members of XIAP pathway [*Cyclin Dependent Kinase Inhibitor 1C* (*CDKN1C*), *Cell division cycle 45* (*CDC45*), *Cyclin Dependent Kinase Inhibitor 2D* (*CDKN2D*)] and the member of the OSR pathway [*Minichromosome Maintenance Complex Component 3* (*MCM3*)] in Her2; and the members of OSR pathways [*Tumor Protein P63* (*TP63*) and *Chromatin Licensing And DNA Replication Factor 1* (*CDT1*)] in LumA breast cancer, and the member of NFkB pathway, *MYC* in Lum A breast cancer (Table 3).

**Table 3:** DE-SN associated with patient overall survival in AA only, W only, or both AA and W.

	AA	W	AA and W
Basal	*ACSM3, COPZ2, MCM5, POLE2, E2F8, BLM* (**XIAP pathway**); *DLL4* (**Jag1-Notch pathway**); *BIRC5* (**NFkB pathway**)	*COPZ2*, *PLAU*, *ABAT* (**XIAP pathway**); *TREM1* (**OSR pathway**), *CCNE2* (**XIAP and OSR pathways**)	*COPZ2* (**XIAP pathway**)
Her2	*ACSM3*, *MCM10*, *GPR137B*, *CDC45*, *LIG1*, *SLC1A4*, *PLAU*, *HIST1H2BJ*, *CDKN2D*, *CDKN1C*, *LEF1*, *SLC14A1* (**XIAP pathway**); *MCM3*, *CCN3*, *PTGS2*, *SCD5*, *NRG1*, *NAP1L3*, *TXNIP* (**OSR pathway**); *NUDT1* (**Immune pathway**)	*LIG1*, *LEF1* (**XIAP pathway);** *CD72* (**TGFβ pathway**); *SCD5*, *CEBPD* (**OSR pathway**)	*LIG1*, *LEF1* (**XIAP pathway**); *SCD5* (**OSR pathway**)
LumB	*MYCBP2*, *STAT4* (**Immune pathway**); *LIPG*, *CDKN1C*, *KLRC1* (**XIAP pathway**); *FGF2 ABCA6* (**OSR pathway**)	*PLAU*, *NRN1*, *ZFPM2* (**XIAP pathway**); *NAP1L3* (**OSR pathway**); *DAB2* (**TGFβ pathway**)	-
LumA	*MCM10*, *LIG1*, *FOSB*, *PIMREG*, *GINS2*, *ABAT* (**XIAP pathway**); *METTL7A*, *TP63*, *MCM4*, *CDT1*, *ANXA3* (**OSR pathway**); *MYC*, *BIRC5* (**NFkB pathway**); *MELK* (**Immune pathway**)	-	-
Normal-like	*FOSB* (**XIAP pathway**)	-	-

DE-SN associated with breast cancer patient overall survival among AA patients only, W patients only, or both AA and W patients. The pathway, to which the ASR genes belong, are shown in parentheses and are bolded.

## Discussion

Disproportionate rates of incidence, metastatic progression and poor survival outcomes are associated with aggressive subtypes of breast cancer in self-identified African American women^2,3^. Much of this disparity in clinical outcome among African American patients with advanced breast cancer remains after controlling for medical coverage, diagnosis, and treatment access^16,38–42^. This suggests that additional societal-level, neighborhood-level and institutional-level, and possibly individual-level, factors contribute to their poorer prognosis. Furthermore, multiple epidemiological studies identify distinct non-genetic risk factors in AA women that induce accumulation of inflammatory and oxidative factors leading to chronic stress microenvironment^2,7,11,13,16^. Tumor cells co-opt anti-apoptotic mechanisms, a hallmark of cancer^43^, to rapidly adapt to microenvironmental and therapeutic stress stimuli that can lead to clonal evolution of death resistant populations^44^. A recent retrospective analysis of a large cohort of non-metastatic, non-IBC, primary invasive breast cancer samples for the apoptotic regulator, XIAP, revealed that *XIAP* mRNA expression is independently associated with poor outcomes and lower pathological complete response (pCR) to anthracycline-bsed neoadjuvant chemotherapy^45^. Based on these clinical observations and our previous preclinical studies identifying ASR pathways linking mitogen activated ser/thr kinase (MNK), X-linked inhibitor of anti-apoptotic protein (XIAP), and nuclear transcription factor (NFκB)-mediated proliferative, invasive, and immunosuppressive breast tumor phenotype^27,30,46^, the present study investigated the expression of these ASR gene sets in TCGA breast cancer samples to identify differentially expressed genes in the PAM50 subtypes and for which there are observed disparities, between AA and W breast cancer patients. We report herein that out of the 226 genes, which can be grouped into three overarching biological processes or signaling axes (XIAP-MNK-NFκB, immune, or TGFβ related), 69.4% were differentially expressed with fold-change > 2 and adjusted *p*-value < 0.05 among the breast cancer subtypes compared to Normal-adjacent samples, or in comparison of subtypes to each other.

Our comparative analysis of the ASR gene sets in TCGA samples stratified by AA and W race identified 29 race-related differentially expressed ASR genes, all of which play a role in cancer biology. For example, *C-X-C Motif Chemokine Ligand 8* (*CXCL8*), reported to promote breast cancer progression^47^, in our analysis was significantly decreased (3-fold-change) among AA patients compared with W patients of Her2 subtype, *MBNL2* (member of the OSR pathway), *TMC6* (member of the immune pathway/Table 1), *PCDH7* (member of the XIAP pathway), and *ACSM3* (member of the XIAP pathway) to be of significance in AA-Basal subtype patients. Interestingly, MBNL2 has been recently reported to control hypoxia response in breast cancer cells and PCDH7 was reported to induce bone metastasis of breast cancer cells^48,49^.

In conjunction, race-stratified survival analysis identified the association of a set of DE-SN (such as *CDKN1C, CDKN2D*, *TP63*, *STAT4*, *MYC*, and *MYCBP2)* with known functions in oncogenic pathways to be distinct in AA or W patient samples, which strongly highlights the importance of stratifying tumors by patient race in survival outcomes. Notably, score analysis for the ASR pathways identified the OSR gene sets score were amplified in advanced breast cancer subtypes and in tumors from AA patients. The GO analysis of differentially expressed ASR genes between tumor and normal breast cancer samples from AAs or Ws reveal race-related molecular pathways. For example, our results suggest that dysregulation of cell death, the ERK1 ERK2 cascade, and the epithelial cell migration processes in Her2 subtype breast cancers in AA but not in W breast cancers has potentially significant implications for treatment approaches. Likewise, these findings suggest that targeting these pathways could achieve different responses when breast cancers of AAs or Ws are treated with similar drugs.

These datasets provide a molecular basis for the epidemiological findings that AA patients’ breast tumors exhibit higher oxidative stress markers compared to W patients^50,51^. Therefore, understanding the underlying biology of aggressive breast cancer subtypes and variants, wherein race- and/or ancestry-related disparities exist in incidence, treatment, and survival outcomes, has the potential to aid in development of new biomarkers and treatment strategies to mitigate these disparities.

Recently, Carrot-Zhang et al. have reported estimated global ancestry for 10,678 patients across 33 cancer types in TCGA^52^. Therefore, we compared the self-identified race and the estimated global ancestry for all the patients from whom we analyzed samples in this study. Our analysis reveals that the self-identified race and estimated global ancestry of the patients to be largely concordant (Supplementary Fig. 2). Therefore, it is possible that ancestry-related individual-level differences and differences in allostatic load also contribute to the differences in ASR genes that we have identified and found to be associated with breast cancer subtype and survival. To further understand the determinants of health underlying differences in ASR genes associated with breast cancer subtype and survival, future studies should focus on estimating local ancestry of chromosomal regions of ASR genes and assessing association with breast cancer subtype and survival.

Although TCGA has a larger number of breast cancer samples from AA patients than for other cancers, a major challenge is the limited number of breast cancer datasets available from AA patients after sorting for PAM50 subtype, with just 6 samples that are designated Normal, rendering limited power for race-related comparative differential gene expression analysis, and eclipsing any potential differences in survival between breast cancer patients of different races or ancestries. Mechanistic studies of ASR genes in breast cancer are ongoing along with the understanding that larger independent cohorts with samples annotated for societal-level, neighborhood-level and institutional-level determinants of health are needed to identify and validate biomarkers.

## Methods

### Datasets and race assignment

The results shown here are in whole based upon data generated by the TCGA Research Network: https://www.cancer.gov/tcga, which are publicly available with prior patient’s consent and institutional review board agreements in place from original authors. The TCGA RNAseq raw counts data from TCGA repository was downloaded (June 15, 2021) using TCGAbiolinks R package (version 2.16.4)^53^. The downloaded RNAseq raw count expression data were normalized for RNAseq library size and dispersion utilizing Limma R package (version 3.44.3)^54,55^. The following multivariate experimental design was employed: sample definition (primary solid tumor, metastatic and solid tissue normal), PAM50 subtype (Basal, Her2, LumB, LumA, and Normal-like), self-identified race of the patient (African American (AA), White (W), Asian, Alaska native American and not reported). Note that despite being described as Normal-like, the Normal-like subtype include tumor samples, not Normal-adjacent.

### Gene expression analysis

A total of 226 genes (Table 1) in the ASR gene sets, which included 101 XIAP-related, 11 NFκB targets, 10 MNK targets, 33 Oxidative Stress Response (OSR)-related, 13 TGFβ-related, 6 JAG1-Notch targets and 52 immune-related, in addition to 14 genes that belong to more than one ASR set, were submitted for comparative analysis using the TCGA breast invasive carcinoma expression dataset. Differential gene expression analysis was performed by applying the linear model of weighted or generalized least squares for series of arrays in limma, and the adjusted *p*-value was calculated using Benjamini-Hochberg method, as described previously^56^. Using R random function, set.seed(1991), we selected 226 random genes to perform the same analysis as a control.

### Gene ontology enrichment analysis

The gene ontology (GO) terms were queried using biomaRt R package (version 2.45.8) as described^57^. The GO terms were matched to the gene in TCGA expression datasets using ensemble identification names. Using the GO terms, we performed enrichment analysis by topGO (version 2.40.0) and org.Hs.eg.db (version 3.11.4) R packages^58,59^. For this analysis, we selected ASR genes that demonstrated differential expression, with a differential fold-change >2 as well as adjusted *p*-value < 0.05, between the compared samples. The selected node size in topGo analysis was 10, the algorithm was classic, which tests the over-representation of GO terms within the group of differentially expressed genes, and the statistic test was Kolmogorov–Smirnov^60^. We determined KS < 0.05 as a cutoff for statistical significance. The GO terms were further reduced using rrvgo (version 1.0.2)^61^.

### Survival analysis

The clinical data associated with TCGA expression data were used to perform survival analyses. The vital status and time until death for all patients (including AA, W, Asian and Alaska native American and not reported) belonging to the indicated PAM50 subtype were appropriately annotated and fitted into Cox Proportional-Hazards Model. The survival R package (version 3.2.11)^62^ was used to compute the hazard ratio per unit and *p*-value of a given differentially expressed ASR gene using Breslow method for the maximum likelihood estimator for the cumulative baseline hazard function. Kaplan–Meier plots for a given differentially expressed ASR gene were generated by comparing the survival probability of the 75th or 25th percentile, with patients grouped by the expression level of the differentially expressed ASR gene. The Kaplan–Meier plots were generated using survminer R package (version 0.4.9)^63^.

### Analysis of gene signature scores

To analyze the score of each ASR gene signature (XIAP, OSR, Immune, and TGFβ pathways as shown in Table 1), we submitted the gene set of each signature for score analysis using GSVA R package (version 1.36.3)^64^. Based on the reported change of expression, we classified the genes in each gene set into up or downregulated. After calculating the score of each gene signature in each sample, we evaluated the score by breast cancer subtype and by patient race, AA or W. To determine the statistical significance of differences in score among different breast cancer subtypes and between AA and W, we employed the Wilcoxon signed-rank test.

### Data analysis

We handled the data and performed analyses using R. Rstudio was used as an interface for R. The following packages: tidyverse, SummarizedExperiment (version 1.18.2), plyr, dplyr, DT, VennDiagram, ggrepel, cowplot, and ggplot2 were also used for data analysis and visualization.

### Reporting summary

Further information on research design is available in the Nature Research Reporting Summary linked to this article.

## Supplementary information

Supplementary Figures and References

Supplementary Table1

Supplementary Table 2

Supplementary Table 3

Supplementary Table 4

Supplementary Table 5

Reporting Summary

## Data Availability

The datasets used in this manuscript can be downloaded from TCGA repository. The retrieved data from TCGA repository is available from the corresponding author on reasonable request.

## Code availability

The code for downloading the datasets, normalizing, and performing the differential expression analysis can be found on GitHub (https://github.com/muthalpy/ASRgenes_TCGA).
