# Supplementary material for: Adaptive stress response genes associated with breast cancer subtypes and survival outcomes reveal race-related differences
Source: NPJ Breast Cancer. 2022 Jun 13;8:73. doi: 10.1038/s41523-022-00431-z (PMC9192737; doi:10.1038/s41523-022-00431-z)
Supplement: Supplementary file 1 — Supplementary Figures and References [file 41523_2022_431_MOESM1_ESM.pdf]

Supplementary Figure 1

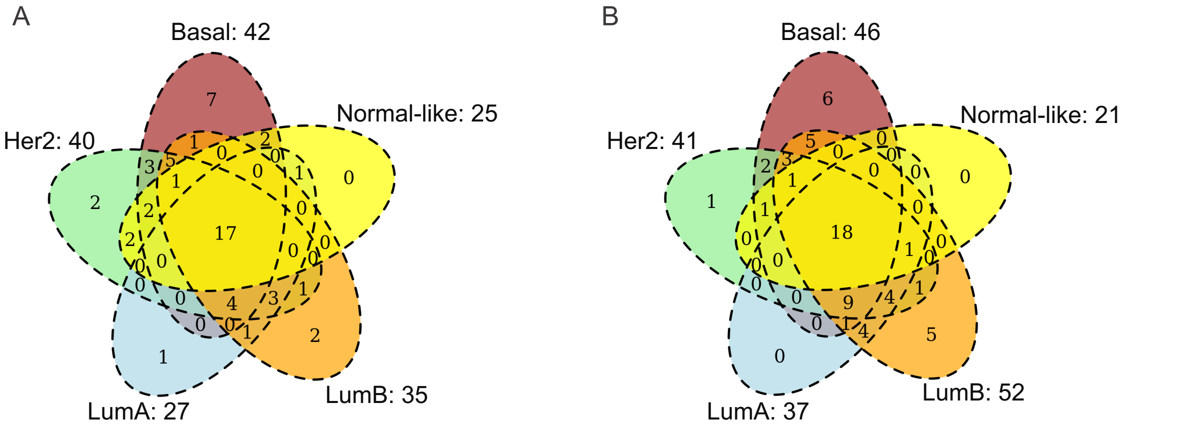

Subtypes vs Normal-adjacent (upregulated DE-SN)

Subtypes vs Normal-adjacent (downregulated DE-SN)

Supplementary Figure 1. Intersection among upregulated or downregulated DE-SN. Venn diagrams depicting the common upregulated DE-SN (**A**) or downregulated DE-SN (**B**) after comparing PAM50 subtypes to Normal.

Supplementary Figure 2

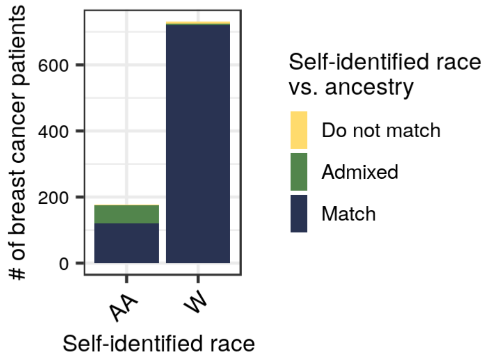

Supplementary Figure 2. Concordance between self-identified race and estimated global ancestry for TCGA breast cancer patients. Bar plot demonstrating the proportion of breast cancer samples in TCGA that we used in our study and whose self-identified race, matches, do not match, or admixed the annotated global ancestry.

Supplementary References

TR1. Mendoza-Villanueva D, Balamurugan K, Ali HR, Kim SR, Sharan S, Johnson RC*, et al.* The C/EBPdelta protein is stabilized by estrogen receptor alpha activity, inhibits SNAI2 expression and associates with good prognosis in breast cancer. Oncogene **2016**;35:6166-76

TR2. Pei H, Hu W, Guo Z, Chen H, Ma J, Mao W*, et al.* Long Noncoding RNA CRYBG3 Blocks Cytokinesis by Directly Binding G-Actin. Cancer Res **2018**;78:4563-72

TR3. Zhao H, Zhang L, Zhang Y, Zhao L, Wan Q, Wang B*, et al.* Calmodulin promotes matrix metalloproteinase 9 production and cell migration by inhibiting the ubiquitination and degradation of TBC1D3 oncoprotein in human breast cancer cells. Oncotarget **2017**;8:36383-98

TR4. Singh JK, Simoes BM, Howell SJ, Farnie G, Clarke RB. Recent advances reveal IL-8 signaling as a potential key to targeting breast cancer stem cells. Breast Cancer Res **2013**;15:210

TR5. Chen F, Zhang Z, Pu F. Role of stanniocalcin-1 in breast cancer. Oncol Lett **2019**;18:3946-53

TR6. Desmedt C, Ouriaghli FE, Durbecq V, Soree A, Colozza MA, Azambuja E*, et al.* Impact of cyclins E, neutrophil elastase and proteinase 3 expression levels on clinical outcome in primary breast cancer patients. Int J Cancer **2006**;119:2539-45

TR7. Di Franco S, Turdo A, Benfante A, Colorito ML, Gaggianesi M, Apuzzo T*, et al.* DeltaNp63 drives metastasis in breast cancer cells via PI3K/CD44v6 axis. Oncotarget **2016**;7:54157-73

TR8. Badic B, Durand S, El Khoury F, De La Grange P, Gentien D, Simon B*, et al.* Prognostic impact of cancer stem cell markers ABCB1, NEO1 and HIST1H2AE in colorectal cancer. Am J Transl Res **2020**;12:5797-807

TR9. Maine EA, Westcott JM, Prechtl AM, Dang TT, Whitehurst AW, Pearson GW. The cancer-testis antigens SPANX-A/C/D and CTAG2 promote breast cancer invasion. Oncotarget **2016**;7:14708-26

TR10. Wakefield L, Robinson J, Long H, Ibbitt JC, Cooke S, Hurst HC*, et al.* Arylamine N-acetyltransferase 1 expression in breast cancer cell lines: a potential marker in estrogen receptor-positive tumors. Genes Chromosomes Cancer **2008**;47:118-26

TR11. Kannan A, Philley JV, Hertweck KL, Ndetan H, Singh KP, Sivakumar S*, et al.* Cancer Testis Antigen Promotes Triple Negative Breast Cancer Metastasis and is Traceable in the Circulating Extracellular Vesicles. Sci Rep **2019**;9:11632

TR12. Han Z, Wang T, Han S, Chen Y, Chen T, Jia Q*, et al.* Low-expression of TMEM100 is associated with poor prognosis in non-small-cell lung cancer. Am J Transl Res **2017**;9:2567-78

TR13. Giulianelli S, Riggio M, Guillardoy T, Perez Pinero C, Gorostiaga MA, Sequeira G*, et al.* FGF2 induces breast cancer growth through ligand-independent activation and recruitment of ERalpha and PRBDelta4 isoform to MYC regulatory sequences. Int J Cancer **2019**;145:1874-88

TR14. Mahadevappa R, Neves H, Yuen SM, Bai Y, McCrudden CM, Yuen HF*, et al.* The prognostic significance of Cdc6 and Cdt1 in breast cancer. Sci Rep **2017**;7:985

TR15. Chua YL, Ito Y, Pole JC, Newman S, Chin SF, Stein RC*, et al.* The NRG1 gene is frequently silenced by methylation in breast cancers and is a strong candidate for the 8p tumour suppressor gene. Oncogene **2009**;28:4041-52

TR16. Dossus L, Kaaks R, Canzian F, Albanes D, Berndt SI, Boeing H*, et al.* PTGS2 and IL6 genetic variation and risk of breast and prostate cancer: results from the Breast and Prostate Cancer Cohort Consortium (BPC3). Carcinogenesis **2010**;31:455-61

TR17. Dai JB, Zhu B, Lin WJ, Gao HY, Dai H, Zheng L*, et al.* Identification of prognostic significance of BIRC5 in breast cancer using integrative bioinformatics analysis. Biosci Rep **2020**;40

TR18. Hu Y, Wang L, Li Z, Wan Z, Shao M, Wu S*, et al.* Potential Prognostic and Diagnostic Values of CDC6, CDC45, ORC6 and SNHG7 in Colorectal Cancer. Onco Targets Ther **2019**;12:11609-21

TR19. Farmer P, Bonnefoi H, Anderle P, Cameron D, Wirapati P, Becette V*, et al.* A stroma-related gene signature predicts resistance to neoadjuvant chemotherapy in breast cancer. Nat Med **2009**;15:68-74

TR20. Shen YL, Gan Y, Gao HF, Fan YC, Wang Q, Yuan H*, et al.* TNFSF9 exerts an inhibitory effect on hepatocellular carcinoma. J Dig Dis **2017**;18:395-403

TR21. Jiang L, Ren L, Zhang X, Chen H, Chen X, Lin C*, et al.* Overexpression of PIMREG promotes breast cancer aggressiveness via constitutive activation of NF-kappaB signaling. EBioMedicine **2019**;43:188-200

TR22. Taouk G, Hussein O, Zekak M, Abouelghar A, Al-Sarraj Y, Abdelalim EM*, et al.* CD56 expression in breast cancer induces sensitivity to natural killer-mediated cytotoxicity by enhancing the formation of cytotoxic immunological synapse. Sci Rep **2019**;9:8756

TR23. Wang J, Zhang C, Wu Y, He W, Gou X. Identification and analysis of long non-coding RNA related miRNA sponge regulatory network in bladder urothelial carcinoma. Cancer Cell Int **2019**;19:327

TR24. Zhao YF, Li L, Li HJ, Yang FR, Liu ZK, Hu XW*, et al.* LncRNA ZFPM2-AS1 aggravates the malignant development of breast cancer via upregulating JMJD6. Eur Rev Med Pharmacol Sci **2020**;24:11139-47
